# Therapy Induced Genome Chaos: A Novel Mechanism of Rapid Cancer Drug Resistance

**DOI:** 10.3389/fcell.2021.676344

**Published:** 2021-06-10

**Authors:** Jing Christine Ye, Steve Horne, Jack Z. Zhang, Lauren Jackson, Henry H. Heng

**Affiliations:** ^1^The Division of Hematology/Oncology, Department of Internal Medicine, University of Michigan, Ann Arbor, MI, United States; ^2^Center for Molecular Medicine and Genomics, Wayne State University School of Medicine, Detroit, MI, United States; ^3^Department of Pathology, Wayne State University School of Medicine, Detroit, MI, United States

**Keywords:** adaptive therapy, drug resistance, karyotype coding, non-clonal chromosome aberrations, polyploid giant cancer cells, two-phased cancer evolution, genome architecture theory, genome reorganization

## The questions

Drug resistance represents an ultimate challenge for cancer treatment. The initial rationale behind using chemo-treatment was the preferred elimination of fast-growing cancer cells, and the drugs' killing power became the priority. Enormous efforts have been made to develop new types of increasingly potent drugs, improve drug delivery, and test various combinations of therapeutic methods (Heng et al., 2010; Heng, 2015). With the identification of many gene mutations and their molecular pathways, encouraged by the success of using imatinib in treating chronic myeloid leukemia (CML) (Horne et al., 2013), molecular targeting offered a curative solution. Currently, immunotherapy further brings new hopes after the increased disappointment of specific targeting therapy, as the success of imatinib for the chronic phase of CML remains a wonderful exception.

Regardless of the wide array of drug therapies available, drug resistance is a universal reality. Even worse, treatment can promote metastasis and cancer lethality (D'Alterio et al., 2020; Pienta et al., 2020a,b). There are many viewpoints/assumptions to explain the mechanisms of drug resistance: multiple drug resistance, cancer stem cells, cellular adaptation and collaboration, epigenetic regulation, cancer heterogenicity, persistent cancer cells, exosomal non-coding RNAs, acquired immune-resistance, and tumor micro-environments including tumor-stromal cross-talk (Heng et al., 2010; Holohan et al., 2013; Keating et al., 2013; Niero et al., 2014; Restifo et al., 2016; Cho and Kim, 2020; Guo et al., 2020; Shen et al., 2020; Swayden et al., 2020; Bhattacharya et al., 2021). Among them, new gene mutations and, in particular, selection of preexisting variants by drug treatment are the most popular mechanisms of cancer drug resistance (Khong and Restifo, 2002; Aktipis et al., 2011; Heng, 2015, 2019). This explanation fits well with neo-Darwinian evolutionary understanding, where the initial killing power is most important. With fewer surviving cancer cells after treatment comes a longer period for the residual cancer cells to grow back and less opportunity for new gene mutations to emerge and then accumulate. However, this concept fails to explain many puzzling issues: why does the treatment often make cancer more aggressive and ultimately untreatable? why the initial benefit of treatment (reducing cancer size) can quickly be lost when the disease becomes out of control and deadly? Can cancer treatment itself elevate the speed of cancer evolution so that the same treatment can harm patients in the name of killing cancer? To effectively address these questions, a new evolutionary framework is needed to discuss: (1) Can cancer cells go through massive and rapid macroevolution immediately following drug treatment, rather than a slow, gradual stepwise micro-evolution (if yes, what is the built-in genomic mechanism for rapid evolution)? (2) Are cancer cells capable of actively selecting a strategy to fight back rather than passively being selected by the treatment (if yes, can smart treatment strategies be developed to eliminate cancer's choice)? Answering these questions also can examine many predictions from some non-neo-Darwinian theories (Shapiro and Noble, 2020).

## Genome Architecture Theory Offers Explanations

The pattern of cancer evolution is rather different from the neo-Darwinian prediction. This realization comes from watching cancer evolution in action experiments (Heng et al., 2006, 2008). By tracing karyotype progression in an immortalization model, the cellular phenotype (growth status, cellular crisis, and population growth), genotype [both clonal chromosome aberrations (CCAs), and non-clonal chromosome aberrations (NCCAs)], were compared longitudinally. These experiments covered main phase transitions of spontaneous immortalization and drug resistance (before, during, and after). What resulted were many surprising observations: 1. Cancer evolution is a two-phased process: karyotype change-mediated punctuated macroevolution and gene mutation-mediated microevolution; 2. The phase transition is triggered by the high level of stress and system instability; and 3. Various NCCAs are important for cancer evolution, and can be used as an index for measuring internal and induced CIN.

As these initial observations were obtained from *in vitro* models (it is challenging to watch evolution in action using an *in vivo* model), extra cautions were taken to explain their biological meanings. First, despite some differences comparing to *in vivo* models, culture cells are truly biological systems, which follow the laws of somatic evolution. Second, the above observations have been confirmed by various *in vitro* and *in vivo* models of different types of cancers and patient samples (Heng, 2019). Third, the two-phased evolution model explains organismal evolution, solving many confusions. Fourth, there is an increased realization that cancer evolution does not fit with neo-Darwinian predictions (Pisco et al., 2013; Ling et al., 2015; Pellestor and Gatinois, 2020; Shapiro and Noble, 2020). Fifth, drastic genomic changes (e.g., big bang, genome chaos) followed by gene mutations have been observed in the majority of cancer types, illustrated by current cancer genome projects (Navin et al., 2011; Sottoriva et al., 2015) (Table 1). Sixth, metastasis is also being linked to chromosome-mediated macroevolution, supporting our prediction (Bloomfield and Duesberg, 2016; Gao et al., 2016; Bakhoum et al., 2018). Finally, a new type of inheritance above individual genes, called karyotype coded system inheritance, has been introduced to illustrate why karyotype change is of ultimate importance in somatic evolution. System inheritance, which organizes the genetic interaction network, is coded by the physical locations of genes and structural elements along and between chromosomes of a given species (Heng, 2019; Ye et al., 2019a).

**Table 1:** Explanations of key concepts, terminologies, and original observations.

Genome chaos
a. Definition: Genome chaos, a process of complex, rapid genome re-organization, leads to the formation of new genomes with altered karyotypes. It was originally described by karyotype analyses in 2006 and later confirmed by sequencing data (Heng et al., 2008; Liu et al., 2014). Chaos, as a behavior of complex adaptive systems, does not simply mean random disorder. Since different types of triggers can reliably initiate the same cellular emergence mechanism, the initiation of genome chaos is likely a programmed and non-random process. However, the stressors and resulting chromosomal variation subtypes are stochastic: many different molecular mechanisms can achieve a state of genome reorganization within a selective evolutionary context (Heng and Heng, 2020). b. Mechanism: Various stress conditions can trigger genome chaos. Genome chaos acts as an evolutionary survival mechanism under crisis. When the reorganization of the genome creates new karyotype coding, new genomic information, the system inheritance, is created, which provides the precondition for macroevolution (Horne et al., 2014; Heng, 2015, 2019; Ye et al., 2019a). c. Biological significance: Genome chaos represents an effective means for creating new genomic information essential for evolution (both cellular adaptation and organismal speciation). Drug treatment-induced drug resistance represents a good example. PGCCs, which are a numerical chaotic genome subtype, can be effectively induced by drugs, and the rapidly reorganized genomes become new systems that are no longer drug-sensitive. d. Genome chaos vs. other types of genetic variations: It is increasingly appreciated that many solid tumors display a significant proportion of triploid or tetraploid karyotypes, offering diverse evolutionary potential. Drug-induced PGCCs, which are not present in primary tumors, often display much higher chromosomal numbers than tetraploid. The key to understanding the power of PGCCs is not just their number of chromosomes, but the capability of creating new karyotypes through the chaotic genome reorganization mechanisms. By rapidly bursting into small cells with different karyotypes, they can quickly deliver macroevolutionary success by providing survivable karyotypes. PGCCs also can produce tetraploid karyotypes. Karyotype chaos has a rather complicated relationship with genetic changes at the SNV/indel level. While in general, karyotype chaos often occurs during the macroevolutionary phase, and gene-level changes dominate the microevolutionary phase, genome chaos also can lead to changes at the gene level. For example, massive chromosomal rearrangement can produce many new fusion genes. Sequencing data also show the burst of gene mutation or copy number variations during evolution. Further studies are needed to co-map different types of genomic alterations within the two-phased cancer evolution. Accordingly, the above understanding will shine new light on diverse mechanisms of cancer drug resistance. Even though drug resistance comes in many flavors, the time is ripe to pay attention to treatment-induced drug resistance and to compare different mechanisms (some of which co-exist) via the lens of two-phased cancer evolution. It is perhaps more important for the cancer research community to consider the realization that many gene-based mechanisms, such as gene amplification, multidrug resistance, and non-genetic resistant mechanisms (Bell and Gilan, 2020), are responsible for drug resistance mainly developed within the microevolutionary phase. By avoiding triggering the transition to the macroevolutionary phase, a treatment window can be created to moderately regulate cellular cancer populations (rather than maximal killing that leads to macroevolution via genome chaos). Such a new frontier will lead to a truly balanced approach to fight cancer drug resistance.
Two phased cancer evolution
a. In contrast to the traditional views of the cancer evolution where stepwise clonal expansion dominates, the two-phased cancer evolutionary model divides cancer evolution into two-phase cycles, each of which is comprised of a karyotype alteration-mediated punctuated macroevolutionary phase, followed by a stepwise gene mutation-mediated microevolutionary phase. Each phase transition is co-mapped with a cellular crisis. Different types of cellular phase transitions, including immortalization, transformation, metastasis, and drug resistance, follow the same pattern of two-phased evolution, although the genotypes of their end products are highly stochastic. From an informational point of view, macroevolution is about new genomic information creation; microevolution is about genomic information maintenance during population growth and information modification (by modifying the gene landscape). b. The two-phased evolutionary pattern can explain organismal evolution well. The punctuated macroevolutionary phase is responsible for creating new systems (e.g., speciation events), while the microevolutionary phase is responsible for the population growth of the novel species.
Adaptive therapy
Adaptive therapy is a treatment strategy that aims to control cancer growth by adjusting treatment options based on evolutionary information (Gatenby et al., 2009; Strobl et al., 2020). The idea was conceptualized based on the ecological consideration that cancer drug resistance could be reduced by leveraging intra-tumoral competition between drug-sensitive and resistant cells. Rather than focus on maximally killing cancer cells, the rationale is to maintain a controllable stable tumor burden by allowing a sizable population of treatment-sensitive cells to survive, which can suppress the growth of the less-fit resistant populations. It should be noted that based on two-phased cancer evolution, the mechanism of how adaptive therapy works could be explained by an alternative idea: moderate drug treatment reduces drug-induced genome chaos, which is responsible for rapid and massive drug resistance. Therefore, moderate killing power can constrain cancer growth without triggering genome chaos-mediated macroevolution. If this explanation is correct, new strategies should be examined using a well-controlled evolutionary strategy for drug treatment, such as selecting specific phases of evolution for treatment, as well as considering the dosage of drugs.
References for initial hypotheses and original observations
a. Genome chaos: PMID: 16688757; PMID: 18936532; PMID: 21215367; PMID: 23571381; PMID: 23622249; PMID: 24299711 b. Genome architecture theory (or genome theory): PMID: 18936532; PMID: 19334004; PMID: 21640814 c. Two-phased cancer evolution: PMID: 16688757; PMID: 19115235; PMID: 21399628; PMID: 25665006 d. PGCCs: PMID: 16314119; PMID: 16948503; PMID: 18936532; PMID: 23524583; PMID: 23571381; PMID: 27991913; PMID: 28436947

Together, a mechanism of drug-induced rapid and massive resistance can be explained by stress-induced macroevolution. To validate this explanation, different chemo-drugs, representing different killing strategies, and different cancer models representing different cancer types and with different degrees of CIN, were used to investigate the mechanism of drug resistance. The following are some conclusions:

1. Different drugs (chemo-drug or target-specific drug), regardless of their mechanisms (e.g., targeting DNA replication or cell dividing machinery), can all eliminate many cancer cells. At the same time, these can also induce genome chaos, a rapid and massive process for re-organizing the genomes, resulting in the emergence of resistant clones with altered karyotypes following a few weeks in culture.
2. The common feature for the survivor cells was the altered karyotype and transcriptome profiles. Initially, the treatment-induced chaotic genomes are highly dynamic, coupled with massive cell death. Later on, many survivor cells are relatively stable, reflected by clonal karyotypes. Comparing the products of multiple runs of parallel experiments resulted in survivor cells that shared similar phenotypes but displayed different karyotypes.
3. The highly diverse types of chaotic genomes detected immediately following drug treatment indicate that different mechanisms can contribute to genome re-organization including structural and numerical genome chaos (Heng et al., 2013; Liu et al., 2014).
4. Despite that it took a much longer time to detect genome chaos in the immortalization model than in the drug-resistant model, the overall pattern was rather similar: high-level stress triggers genome chaos; leading to genome re-organization and macroevolution, followed by microevolution. Rapid drug resistance is not mainly caused by preexisting gene mutation, but drug treatment-induced new cellular species (see Table 1 for more information regarding the two-phased cancer evolution).

## Polyploid Giant Cancer Cells: the New Kid on the Block

The clinical implication of treatment-induced resistance is highly significant. However, the research community has paid little attention to it as “these induced chaotic genomes are too extraordinary to be true, they must be non-survivable thus not important” (from an anonymous reviewer).

Despite that drug treatment-induced karyotype chaos mediated rapid resistance was previously reported (Heng et al., 2008), without the Genome Theory, and equally important, without the lesson from the cancer genome project where the genomic landscape of cancer is highly dynamic and hard to target, the field was not ready to accept the new mechanism of drug resistance.

Fortunately, increased sequencing data has forcefully supported the importance of large-scale genome re-organization in cancer evolution, albeit using different terms such as chromothripsis, chromoplexy, chromoanagenesis and Chromohelkosis to describe different subtypes of genome chaos (Stephens et al., 2011; Baca et al., 2013; Iourov et al., 2020; Pellestor and Gatinois, 2020). Then, interest emerged in the field of polyploid giant cancer cells (PGCCs).

PGCCs and micronuclei clusters have been classified as numerical NCCAs reflecting the chromosome instability (Heng et al., 2008; Stevens et al., 2011). They were linked to cellular immortalization and drug resistance via treatment-induced numerical genome or karyotype chaos (Heng et al., 2006, 2013; Liu et al., 2014). It was also observed that some of the giant cells can generate viable cells (Erenpreisa et al., 2005, 2020). The process can be linked to senescence, polyploidy, and newly generated mitotic cells (Walen, 2005). The exciting mechanisms of PGCCs in chemoresistance have become clear. By reviewing the long-ignored history of the PGCCs in laboratory observations, PGCCs have been purified and cultured from cancer cells for characterizations, which illustrated the dynamic relationship among stress, endoreduplication, cell fusion, budded or burst cells, stemness, cancer evolutionary potential, and chemoresistance (Zhang et al., 2014). Furthermore, it was concluded that PGCCs may represent a fundamental mechanism to initiate genome reorganization to generate new cells in response to chemotherapy-induced stress and rapid drug resistance (Niu et al., 2016), agreeing with the observation that harsh treatment can induce genome chaos mediated drug-resistant phenotypes (Heng et al., 2008; Heng, 2015). Finally, following the characterization of “the giant cell cycle,” and the comparison of PGCCs with blastomeres, the relationship between PGCCs, different types of cancers, and developmental stages/hierarchy were synthesized, which laid out the strong basis for PGCC studies (Niu et al., 2017; Liu, 2018, 2020). Meanwhile, an exciting frontier of using PGCCs to study drug resistance has emerged, involving diverse molecular mechanisms (Mirzayans et al., 2018; Lin et al., 2019; Herbein and Nehme, 2020; Mannan et al., 2020; Pienta et al., 2020b; Tagal and Roth, 2020; White-Gilbertson and Voelkel-Johnson, 2020).

## The Crisis Created New Karyotype Information: Mechanism of Treatment-Induced Macroevolution

Many individual molecular mechanisms are identified to explain different ways of producing chaotic genomes including chromothripsis, chromoplexy, and PGCCs, based on neo-Darwinian's gene-centric, microevolutionary principles. According to the evolutionary mechanism of cancer, many specific molecular pathways can be linked to stress-caused genome dynamics. However, the evolutionary significance, perhaps, is not about any given subtype(s) of the chaotic genome, but rather the consequence of this stress-induced process to create new genomes with new genomic information. For example, these drug-induced giant cells are unstable, which can generate drug-resistant cell populations with re-organized genomes. The general model that integrates “high stress in crisis,” “chaotic genome like giant cell,” “new karyotypes,” and “drug-resistance” can be described as a three-stage-process based on two-phased cancer evolution: (1) Under high stresses with cell-killing power, a conservative developmental-related process is active leading to polyploidization. (2) The self-organization process generates large numbers of smaller cells with new genomes selected by genome chaos mediated macroevolution. (3) The survivor cells can further grow into dominant populations via microevolution, often with help of diverse cancer genes. In other words, high stress is the trigger factor, giant cells are the transitional phenotype, genome chaos is the process for rapid and massive genome re-organization, and the newly formed karyotypes of the survivor cell populations are the genotype of drug resistance. Alternatively, some cells can directly re-organize their genome without going through the polyploidization process. All roads lead to new information creation. This explanation fits well with Genome Architecture Theory where karyotype codes system inheritance, and re-organization of the genome represents a powerful way to generate new genomic information essential for cancer macroevolution. The formed cancer systems could grow into dominant cell populations with the help of gene mutation/epigenetic alteration-mediated microevolution (Heng, 2009, 2019; Ye et al., 2019a,b; Heng and Heng, 2020). A recent report that links genome chaos and then gene amplification in drug resistance can be better explained by the above two-phased model (Shoshani et al., 2021).

## Actions

The above understanding has diagnosed the conceptual limitations of current treatment strategies, which are mainly focused on killing cancer cells based on the stepwise microevolution cancer model. Knowing that drug treatment can paradoxically harm patients by promoting cancer macroevolution through the creation of new karyotypes, urgent actions are needed for the following priorities: First, validation that drug-induced genome chaos-mediated drug resistance is common in the clinical setting. It is important to know to what extent can this phenomenon be avoided by modifying treatment strategies. Second, we hypothesize that reduced genome chaos might be the reason behind the success of adaptive therapy (Gatenby et al., 2009; Heng and Heng, 2020). We further anticipate that by using lower dosage specifically within the microevolutionary phase, adaptive therapy will have better results. Third, as a high level of chromosomal structural abnormality can suppress the immune response to tumor cells, it is important to investigate if treatment-induced genome chaos represents a potential mechanism of escape from immune therapy. Finally, accepting two-phased active cancer evolution (not just passive selection) and applying different diagnostic and treatment strategies accordingly holds the key for future therapeutic success (Heng, 2015, 2019; Shapiro and Noble, 2020).

## Author Contributions

JY and HH drafted the manuscript. All authors participated in the discussion, literature search, and editing of the manuscript.

## Conflict of Interest

The authors declare that the research was conducted in the absence of any commercial or financial relationships that could be construed as a potential conflict of interest.
